# Crystal structure and Hirshfeld surface analysis of di-μ-chlorido-bis­[(aceto­nitrile-κ*N*)chlorido­(ethyl 5-methyl-1*H*-pyrazole-3-carboxyl­ate-κ^2^ *N* ^2^,*O*)copper(II)]

**DOI:** 10.1107/S2056989021010653

**Published:** 2021-10-26

**Authors:** Oleksandr S. Vynohradov, Vadim A. Pavlenko, Olesia I. Kucheriv, Irina A. Golenya, Denys Petlovanyi, Sergiu Shova

**Affiliations:** a Department of Chemistry, Taras Shevchenko National University of Kyiv, Volodymyrska str. 64/13, 01601 Kyiv, Ukraine; b Enamine Ltd, Oleksandra Matrosova Str. 23, Kyiv 01103, Ukraine; c "Poni Petru" Institute of Macromolecular Chemistry, Aleea Gr. Ghica, Voda 41A, 700487 Iasi, Romania

**Keywords:** copper, copper complexes, crystal structure, pyrazole, X-ray crystallography, Hirshfeld surface analysis, one-pot reaction, direct synthesis, oxidative dissolution

## Abstract

The title compound, [Cu_2_Cl_4_(C_5_H_10_N_2_O_2_)_2_(CH_3_CN)_2_] or [Cu_2_(μ_2_-Cl)_2_(CH_3_—Pz-COOCH_2_CH_3_)_2_Cl_2_(CH_3_CN)_2_], was synthesized using a one-pot reaction of copper powder, copper(II) chloride dihydrate and ethyl 5-methyl-1*H*-pyrazole-3-carboxyl­ate (CH_3_—Pz-COOCH_2_CH_3_) in aceto­nitrile under ambient conditions. This complex consists of discrete binuclear mol­ecules with a {Cu_2_(μ_2_-Cl)_2_} core, in which the Cu⋯Cu distance is 3.8002 (7) Å. The pyrazole-based ligands are bidentate coordinated, leading to the formation of two five-membered chelate rings. The coordination geometry of both copper atoms (ON_2_Cl_3_) can be described as distorted octa­hedral on account of the aceto­nitrile coordination. A Hirshfeld surface analysis suggests that the most important contributions to the surface contacts are from H⋯H (40%), H⋯Cl/Cl⋯H (24.3%), H⋯O/O⋯H (11.8%), H⋯C/C⋯H (9.2%) and H⋯N/N⋯H (8.3%) inter­actions.

## Abstract 2

The title compound, [Cu_2_Cl_4_(C_5_H_10_N_2_O_2_)_2_(CH_3_CN)_2_] or [Cu_2_(μ_2_-Cl)_2_(CH_3_—Pz-COOCH_2_CH_3_)_2_Cl_2_(CH_3_CN)_2_], was synthesized using an one-pot reaction of copper powder, copper(II) chloride dihydrate and ethyl 5-methyl-1*H*-pyrazole-3-carboxyl­ate (CH_3_—Pz-COOCH_2_CH_3_) in aceto­nitrile under ambient conditions. This complex consists of discrete binuclear mol­ecules with a {Cu_2_(μ_2_-Cl)_2_} core.

## Chemical context

Pyrazoles can form structures of various nuclearities, ranging from mononuclear (Mighell *et al.*, 1975 ▸; Liu *et al.*, 2001 ▸; Małecka *et al.*, 2003 ▸) to polynuclear complexes (He, 2011 ▸; Contaldi *et al.*, 2009 ▸; Chandrasekhar *et al.*, 2008 ▸) and metallacycles (Vynohradov *et al.*, 2020*a* ▸; Surmann *et al.*, 2016 ▸; Galassi *et al.*, 2012 ▸) with specific mol­ecular topologies. By performing the synthesis of metal complexes by oxidative dissolution of metals, commonly known as direct synthesis (Kokozay *et al.*, 2018 ▸; Plyuta *et al.*, 2020 ▸; Sirenko *et al.*, 2020 ▸; Li *et al.*, 2021 ▸), copper can be introduced in a zerovalent state. Copper powder can be oxidized in solution in the presence of proton-donating agents, such as pyrazoles, to form polynuclear complexes, where two copper atoms are connected by a bidentate-bridging deprotonated pyrazole (Vynohradov *et al.*, 2020*b* ▸; Davydenko *et al.*, 2013 ▸). Many examples of copper coordination compounds have been synthesized and described in which two copper atoms are connected by halogen bridges, for example, through chlorine anions, deprotonated ligand mol­ecules and also hydroxyl groups (Vincent *et al.*, 2018 ▸; Wei *et al.*, 2012 ▸; Mezei *et al.*, 2004 ▸). Copper(II) pyrazolate complexes have attracted considerable inter­est for their inter­esting magnetic properties (Malinkin *et al.*, 2012 ▸; Spodine *et al.*, 1999 ▸) and abilities to bind DNA (Vafaza­deh *et al.*, 2015 ▸; Kulkarni *et al.*, 2011 ▸). Finally, the anti­oxidant (Kupcewicz *et al.*, 2013 ▸) and anti­cancer (Santini *et al.*, 2014 ▸) activities of these compounds should be noted. Relatively few unsymmetrical pyrazole-containing ligands with different chelating arms in the 3- and 5-positions and their coordination compounds have been investigated so far (Konrad *et al.*, 2001 ▸; Dubs *et al.*, 2006 ▸; Krämer *et al.*, 2002 ▸; Röder *et al.*, 2002 ▸; Penkova *et al.*, 2010 ▸). Considering the above, we understand the importance of accumulating a theoretical information base on such coordination compounds, and therefore in this article we report the synthesis, crystal structure and Hirshfeld surface analysis of a new binuclear copper(II) complex with unsymmetrical pyrazole ethyl 5-methyl-1*H*-pyrazole-3-carboxyl­ate – [Cu_2_(μ_2_-Cl)_2_(CH_3_-Pz-COOCH_2_CH_3_)_2_Cl_2_(CH_3_CN)_2_]. 
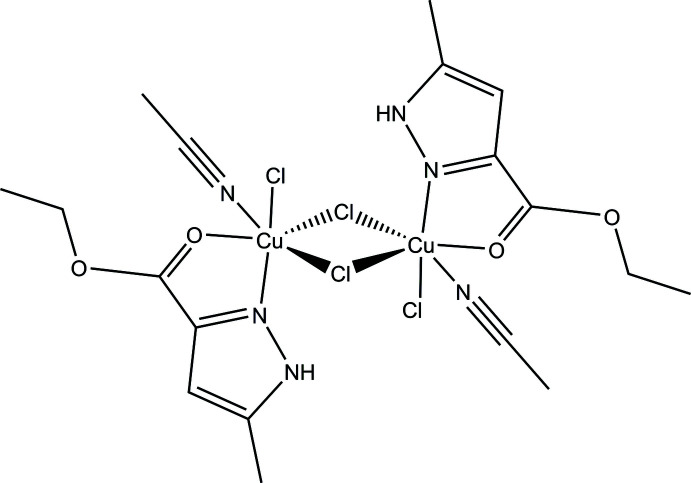


## Structural commentary

**Figure 1:**
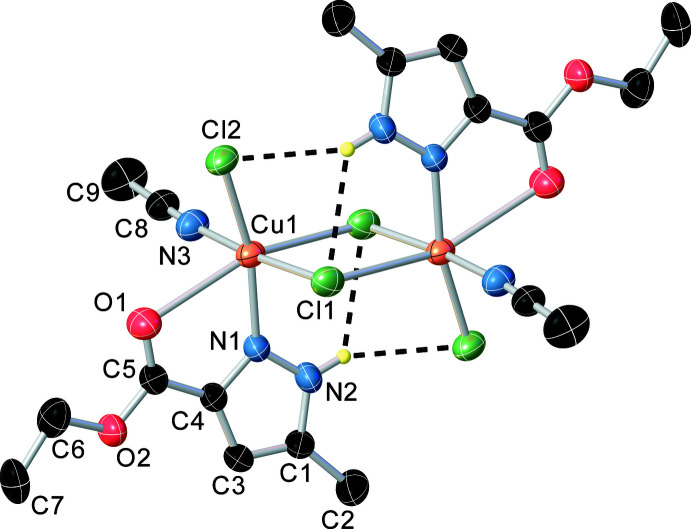
The mol­ecular structure of the title compound, with displacement ellipsoids drawn at the 50% probability level. Irrelevant hydrogen atoms were omitted for clarity.

The title compound (Fig. 1 ▸) is a binuclear cyclic copper(II) pyrazole-containing complex which crystallized in the monoclinic *P*2_1_/*c* space group. The asymmetric unit consists of one copper ion, one ethyl 5-methyl-1*H*-pyrazole-3-carboxyl­ate ligand, one coord­inated aceto­nitrile mol­ecule and two chlorine ions. One of these chlorine ions bridges two metal centers, thus connecting two symmetry-generated fragments. The structure of this complex can be described as a dimer of formula [CuCl_2_(C_7_H_10_N_2_O_2_)(CH_3_CN)]_2_ in which the CH_3_-Pz-COOCH_2_CH_3_ ligand is coordinated in a bidentate way and remains protonated. The copper atom has a distorted octa­hedral coordination environment formed by three chlorine atoms, one nitro­gen atom of the aceto­nitrile mol­ecule and two atoms of the unsymmetrical pyrazole ligand – the pyridine-like N1 atom and atom O 1 of the ester substituent in position 3 of the pyrazole ring. The bidentate coordination of the pyrazole ligand leads to the formation of a five-membered chelate ring. The atoms in the ring deviate only slightly from planarity [the Cu1 atom is out of the Cu1/N1/C4/C5/O1 plane by 0.0222 (8) Å; N1 by −0.0406 (14) Å; C4 by 0.0326 (15) Å; C5 by 0.0031 (18) Å and O1 by −0.0172 (14) Å]. Both the copper atoms and the bridging chlorine atoms lie in the same plane without deviations from planarity. The inter­metallic distance in the dimer unit is 3.8002 (7) Å while the chlorine–chlorine separation in the four-membered bimetallic cycle is 3.5894 (15) Å.

**Figure 2:**
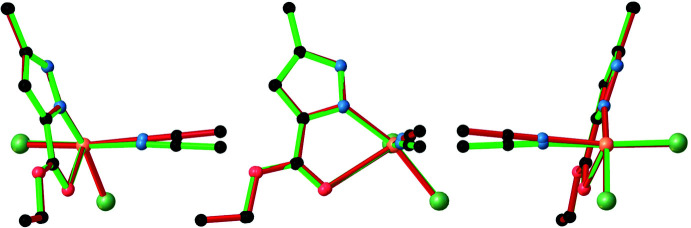
Overlay diagram of the asymmetric units of the structures of the title compound (red) and of a similar complex with methyl 5-methyl-1*H*-pyrazole-3-carboxyl­ate (green) which shows the similarity of the structure regardless of the organic radical *R* in the COO*R* ester group of the substituent on the pyrazole ring.

An overlay of the asymmetric units of the structures of the title compound (red) and a similar complex with methyl 5-methyl-1*H*-pyrazole-3-carboxyl­ate (green) is presented in Fig. 2 ▸. The structures were compared using *OLEX2* software (Dolomanov *et al.*, 2009 ▸). It was found that the structure of the complex does not change regardless of the organic radical *R* in the COO*R* ester group, whether –CH_3_ or –CH_2_—CH_3_. The crystal structures of these compounds are also similar. In addition, the inter­metallic distance in the above structures differs approximately by 0.1 Å and the chlorine–chlorine separation in the four-membered bimetallic ring differs by 0.05 Å [Cu⋯Cu = 3.7047 (7) Å and Cl⋯Cl = 3.5364 (11) Å for the methyl analogue]. The mol­ecular structure is stabilized by intra­molecular N—H⋯Cl and C—H⋯Cl hydrogen bonds (Table 1 ▸).

## Supra­molecular features

**Figure 3:**
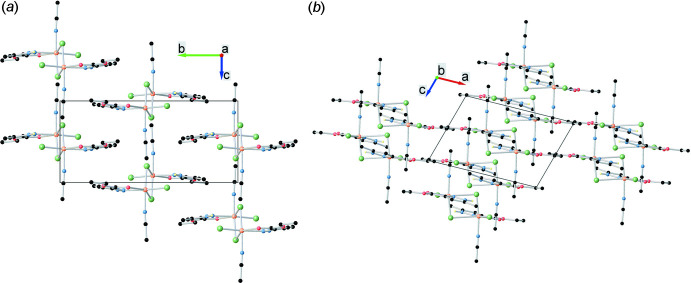
Crystal packing of the title compound viewed along (*a*) the *a*- and (*b*) the *b*-axis directions. Selected hydrogen atoms were omitted for clarity.

The crystal packing of the title compound (Fig. 3 ▸) consists of discrete binuclear mol­ecules with a {Cu_2_(μ_2_-Cl)_2_} core, which form a planar bimetallic ring. The four-membered Cu1/Cl1/Cu1^i^/Cl1^i^ planes of the bimetallic rings are situated perpendicular to the *b* axis, while the chelate ring planes are located approximately parallel. No inter­molecular hydrogen bonds were identified in the crystal structure. The minimum separation between the Cl atoms of neighbouring mol­ecules inside one unit cell is 4.4013 (13) Å for Cl1^i^ and Cl1^ii^ [symmetry codes: (i) 1 − *x*, 1 − *y*, 2 − *z*; (ii) *x*, *y*, −1 + *z*] while the minimum distance between two copper atoms is 7.6498 (3) Å for Cu1 and Cu1^ii^.

## Hirshfeld surface analysis

**Figure 4:**
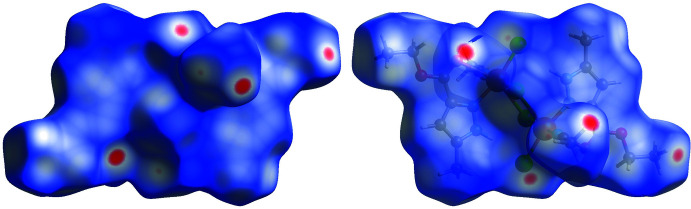
Two projections of Hirshfeld surfaces mapped over d_norm_ showing the inter­molecular inter­actions within the mol­ecule.

**Figure 5:**
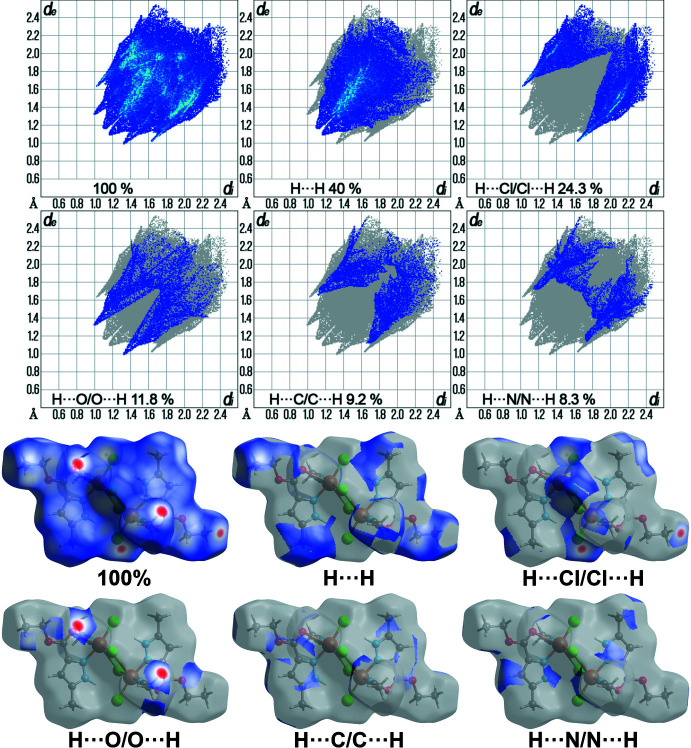
The overall two-dimensional fingerprint plot and those delineated into specified inter­actions. Hirshfeld surface representations with the function *d*_norm_ plotted onto the surface for the different inter­actions.

The Hirshfeld surface analysis and the associated two-dimensional fingerprint plots were performed using *Crystal Explorer 17.5* (Turner *et al.*, 2018 ▸), with a standard resolution of the three-dimensional *d*_norm_ surfaces plotted over a fixed colour scale of −0.1996 (red) to 1.1926 (blue) a.u. The pale-red spots in Fig. 4 ▸ represent short contacts and negative *d*_norm_ values on the surface corresponding to the inter­actions described above. The Hirshfeld surfaces mapped over *d*_norm_ are shown for the H⋯H, H⋯Cl/Cl⋯H, H⋯O/O⋯H, H⋯C/C⋯H and H⋯N/N⋯H contacts, the overall two-dimensional fingerprint plot and the decomposed two-dimensional fingerprint plots are given in Fig. 5 ▸. Twelve short inter­atomic contacts in the range 2.34–2.8 Å are indicated by the faint red spots. Two pairs of inter­molecular C—H⋯O contacts between the O1 atom of the ester substituent and the hydrogen atom of the methyl group of the coordinated aceto­nitrile were the shortest. Also, four inter­molecular C—H⋯Cl contacts with a length of 2.685 Å, which are present between the terminal chlorine atoms and the hydrogen atoms of the ethyl group (hydrogen atom near C7) of the ester substituent are also short. Finally, four inter­molecular C—H⋯Cl contacts with a length of 2.8 Å are observed between the terminal chlorine atoms and the hydrogen atoms of the –CH_3_ group of the aceto­nitrile mol­ecule. For the title compound, the most significant contributions to the overall crystal packing are from H⋯H (40%), H⋯Cl/Cl⋯H (24.3%), H⋯O/O⋯H (11.8%), H⋯C/C⋯H (9.2%) and H⋯N/N⋯H (8.3%) contacts. The small contribution of the other weak inter­molecular C⋯C (2.9%), C⋯O/O⋯C (2.1%), C⋯N/N⋯C (0.8%), C⋯Cl/Cl⋯C (0.3%), O⋯N/N⋯O (0.3%) and Cl⋯Cl (0.1%) contacts has a negligible effect on the packing. In addition, qu­anti­tative physical properties of the Hirshfeld surface for the title compound were obtained, such as mol­ecular volume (657.89 Å^3^), surface area (571.56 Å^2^), globularity (0.640), as well as asphericity (0.147).

## Database survey

Six similar structures are registered in the Cambridge Structural Database (Version 2021.1; Groom *et al.*, 2016 ▸): two reports of complexes with methyl 5-methyl-1*H*-pyrazole-3-carboxyl­ate [UMUXEI (Rheingold, 2021 ▸) and ZEQGUZ (Shakirova *et al.*, 2012 ▸)], two reports of the free ligand ethyl 5-methyl-1*H*-pyrazole-3-carboxyl­ate [FAQSAR01 (Mague *et al.*, 2018 ▸) and FAQSAR02 (Kusakiewicz-Dawid *et al.*, 2019 ▸)] and two structure reports of the same ligand with a different name and cell parameters (Elguero *et al.*, 1999 ▸) [3-eth­oxy­carbonyl-5-methyl­pyrazole (FAQSAR) and 4-bromo-3-eth­oxy­carbonyl-5-methyl­pyrazole (FAQTAS)].

## Synthesis and crystallization

[Cu_2_(μ_2_-Cl)_2_(CH_3_-Pz-COOCH_2_CH_3_)_2_Cl_2_(CH_3_CN)_2_] was synthesized at room temperature by the oxidative dissolution method by the addition of a copper powder (1.56 mmol, 0.1 g) and copper(II) chloride dihydrate (3.1 mmol, 0.53 g) mixture to an aceto­nitrile (9 ml) solution of ethyl 5-methyl-1*H*-pyrazole-3-carboxyl­ate (4.67 mmol, 0.72 g). The mixture was stirred without heating for three h with free air access until dissolution of the copper powder and a green precipitate of the product was obtained. The precipitate was filtered off and re-dissolved in aceto­nitrile. Green crystals suitable for X-ray analysis were obtained by slow evaporation of the solvent. The IR spectra of the starting pyrazole ligand and the obtained green crystals of the title coordination compound are given in the supporting information.

## Refinement

Crystal data, data collection and structure refinement details are summarized in Table 2 ▸. C-bound H atoms were positioned geometrically (C—H = 0.93–0.97) and refined as riding with *U*_iso_(H) = 1.2*U*_eq_(C) or 1.5*U*_eq_(C-meth­yl). N-bound H atoms were refined with *U*_iso_(H) = 1.2*U*_eq_(N).

## Supplementary Material

Crystal structure: contains datablock(s) I. DOI: 10.1107/S2056989021010653/zq2267sup1.cif

Structure factors: contains datablock(s) I. DOI: 10.1107/S2056989021010653/zq2267Isup2.hkl

Supporting information file. DOI: 10.1107/S2056989021010653/zq2267Isup3.cdx

IR spectrum of the title compound. DOI: 10.1107/S2056989021010653/zq2267sup4.txt

IR spectrum of the free ligand. DOI: 10.1107/S2056989021010653/zq2267sup5.txt

CCDC reference: 2115608

Additional supporting information:  crystallographic information; 3D view; checkCIF report

## Figures and Tables

**Table 1:** Hydrogen-bond geometry (Å, °)

*D*—H⋯*A*	*D*—H	H⋯*A*	*D*⋯*A*	*D*—H⋯*A*
N2—H2⋯Cl1^i^	0.80 (2)	2.72 (2)	3.281 (2)	129 (2)
N2—H2⋯Cl2^i^	0.80 (2)	2.59 (2)	3.273 (2)	145 (1)
C7—H7*A*⋯Cl2^ii^	0.96	2.79	3.662 (4)	151

Symmetry codes: (i) -x+1, -y+1, -z+2; (ii) -x, y-{\script{1\over 2}}, -z+{\script{3\over 2}}.

**Table 2:** Experimental details

Crystal data	
Chemical formula	[Cu_2_Cl_4_(C_5_H_10_N_2_O_2_)_2_(C_2_H_3_N)_2_]
*M*_r_	659.33
Crystal system, space group	Monoclinic, *P*2_1_/*c*
Temperature (K)	293
*a*, *b*, *c* (Å)	11.3934 (4), 15.9822 (5), 7.6498 (3)
β (°)	106.226 (4)
*V* (Å^3^)	1337.48 (9)
*Z*	2
Radiation type	Mo *K*α
μ (mm^−1^)	2.03
Crystal size (mm)	0.45 × 0.2 × 0.1

Data collection	
Diffractometer	Rigaku Xcalibur, Eos
Absorption correction	Multi-scan (*CrysAlis PRO*; Rigaku OD, 2021 ▸)
*T*_min_, *T*_max_	0.839, 1.000
No. of measured, independent and observed [*I* > 2σ(*I*)] reflections	9132, 3062, 2380
*R*_int_	0.029
(sin θ/λ)_max_ (Å^−1^)	0.666

Refinement	
*R*[*F* ^2^ > 2σ(*F* ^2^)], *wR*(*F* ^2^), *S*	0.036, 0.083, 1.04
No. of reflections	3062
No. of parameters	158
H-atom treatment	H atoms treated by a mixture of independent and constrained refinement
Δρ_max_, Δρ_min_ (e Å^−3^)	0.30, −0.34

Computer programs: *CrysAlis PRO* (Rigaku OD, 2021 ▸), *SHELXLT* (Sheldrick, 2015*a* ▸), *SHELXL2018/3* (Sheldrick, 2015*b* ▸) and *OLEX2* (Dolomanov *et al.*, 2009 ▸).

## supplementary crystallographic information

### Crystal data

**Table:**

[Cu_2_Cl_4_(C_5_H_10_N_2_O_2_)_2_(C_2_H_3_N)_2_]	*F*(000) = 668
*M**_r_* = 659.33	*D*_x_ = 1.637 Mg m^−^^3^
Monoclinic, *P*2_1_/*c*	Mo *K*α radiation, λ = 0.71073 Å
*a* = 11.3934 (4) Å	Cell parameters from 3607 reflections
*b* = 15.9822 (5) Å	θ = 2.3–27.2°
*c* = 7.6498 (3) Å	µ = 2.03 mm^−^^1^
β = 106.226 (4)°	*T* = 293 K
*V* = 1337.48 (9) Å^3^	Block, green
*Z* = 2	0.45 × 0.2 × 0.1 mm

### Data collection

**Table:**

Rigaku Xcalibur, Eos diffractometer	3062 independent reflections
Radiation source: fine-focus sealed X-ray tube, Enhance (Mo) X-ray Source	2380 reflections with *I* > 2σ(*I*)
Graphite monochromator	*R*_int_ = 0.029
Detector resolution: 16.1593 pixels mm^-1^	θ_max_ = 28.3°, θ_min_ = 1.9°
ω scans	*h* = −14→14
Absorption correction: multi-scan (CrysAlisPro; Rigaku OD, 2021)	*k* = −18→21
*T*_min_ = 0.839, *T*_max_ = 1.000	*l* = −9→9
9132 measured reflections	

### Refinement

**Table:**

Refinement on *F*^2^	0 restraints
Least-squares matrix: full	Hydrogen site location: inferred from neighbouring sites
*R*[*F*^2^ > 2σ(*F*^2^)] = 0.036	H atoms treated by a mixture of independent and constrained refinement
*wR*(*F*^2^) = 0.083	*w* = 1/[σ^2^(*F*_o_^2^) + (0.0338*P*)^2^ + 0.250*P*] where *P* = (*F*_o_^2^ + 2*F*_c_^2^)/3
*S* = 1.04	(Δ/σ)_max_ = 0.001
3062 reflections	Δρ_max_ = 0.30 e Å^−^^3^
158 parameters	Δρ_min_ = −0.34 e Å^−^^3^

### Special details

**Table:**

Geometry. All esds (except the esd in the dihedral angle between two l.s. planes) are estimated using the full covariance matrix. The cell esds are taken into account individually in the estimation of esds in distances, angles and torsion angles; correlations between esds in cell parameters are only used when they are defined by crystal symmetry. An approximate (isotropic) treatment of cell esds is used for estimating esds involving l.s. planes.

### Fractional atomic coordinates and isotropic or equivalent isotropic displacement parameters (Å^2^)

**Table:**

	*x*	*y*	*z*	*U*_iso_*/*U*_eq_	
Cu1	0.32946 (3)	0.47882 (2)	0.91290 (4)	0.03655 (12)	
Cl1	0.45100 (6)	0.48716 (4)	1.20189 (9)	0.04438 (18)	
Cl2	0.23810 (6)	0.60138 (4)	0.93837 (10)	0.04752 (19)	
O1	0.14691 (18)	0.38325 (11)	0.9411 (3)	0.0501 (5)	
O2	0.12145 (16)	0.24499 (11)	0.9610 (3)	0.0471 (5)	
N1	0.37558 (18)	0.35720 (12)	0.9058 (3)	0.0359 (5)	
N2	0.4841 (2)	0.32429 (13)	0.9131 (3)	0.0406 (6)	
H2	0.541 (2)	0.3509 (10)	0.9021 (5)	0.049*	
N3	0.2412 (2)	0.47671 (13)	0.6418 (3)	0.0452 (6)	
C1	0.4879 (2)	0.24100 (15)	0.9412 (4)	0.0414 (7)	
C2	0.6021 (3)	0.19159 (18)	0.9591 (5)	0.0628 (10)	
H2A	0.624715	0.194792	0.847440	0.094*	
H2B	0.588090	0.134232	0.984660	0.094*	
H2C	0.666779	0.214025	1.056683	0.094*	
C3	0.3747 (2)	0.21906 (15)	0.9510 (4)	0.0417 (6)	
H3	0.347635	0.165569	0.967998	0.050*	
C4	0.3075 (2)	0.29279 (15)	0.9307 (3)	0.0356 (6)	
C5	0.1848 (2)	0.31277 (16)	0.9433 (4)	0.0375 (6)	
C6	0.0021 (2)	0.2595 (2)	0.9877 (4)	0.0567 (8)	
H6A	−0.049565	0.289751	0.884815	0.068*	
H6B	0.009748	0.292321	1.096964	0.068*	
C7	−0.0522 (3)	0.1761 (2)	1.0052 (5)	0.0624 (9)	
H7A	−0.071569	0.147755	0.890001	0.094*	
H7B	−0.125387	0.183416	1.042309	0.094*	
H7C	0.005383	0.143312	1.094653	0.094*	
C8	0.1955 (3)	0.48097 (15)	0.4916 (4)	0.0417 (6)	
C9	0.1373 (4)	0.4863 (2)	0.2988 (4)	0.0710 (10)	
H9A	0.150266	0.435166	0.241128	0.107*	
H9B	0.171560	0.532163	0.248413	0.107*	
H9C	0.051142	0.495250	0.278268	0.107*	

### Atomic displacement parameters (Å^2^)

**Table:**

	*U*^11^	*U*^22^	*U*^33^	*U*^12^	*U*^13^	*U*^23^
Cu1	0.0379 (2)	0.03067 (19)	0.0376 (2)	0.00332 (12)	0.00486 (14)	−0.00063 (12)
Cl1	0.0504 (4)	0.0426 (4)	0.0359 (4)	0.0007 (3)	0.0049 (3)	0.0039 (3)
Cl2	0.0448 (4)	0.0378 (4)	0.0559 (5)	0.0095 (3)	0.0074 (3)	−0.0022 (3)
O1	0.0491 (12)	0.0414 (11)	0.0637 (14)	0.0052 (9)	0.0224 (10)	−0.0034 (9)
O2	0.0351 (10)	0.0452 (11)	0.0642 (14)	−0.0029 (8)	0.0190 (9)	0.0025 (9)
N1	0.0305 (11)	0.0312 (11)	0.0449 (14)	−0.0011 (9)	0.0088 (10)	−0.0021 (9)
N2	0.0329 (12)	0.0353 (12)	0.0544 (16)	−0.0048 (9)	0.0133 (11)	−0.0057 (10)
N3	0.0471 (14)	0.0425 (13)	0.0431 (15)	0.0053 (10)	0.0077 (12)	−0.0029 (10)
C1	0.0382 (15)	0.0301 (13)	0.0526 (18)	0.0006 (11)	0.0074 (13)	−0.0075 (11)
C2	0.0442 (17)	0.0463 (17)	0.094 (3)	0.0062 (13)	0.0121 (18)	−0.0150 (16)
C3	0.0399 (15)	0.0311 (13)	0.0517 (18)	−0.0041 (11)	0.0085 (13)	−0.0003 (11)
C4	0.0338 (13)	0.0329 (13)	0.0382 (16)	−0.0032 (10)	0.0069 (11)	−0.0037 (10)
C5	0.0370 (14)	0.0385 (15)	0.0368 (16)	−0.0040 (11)	0.0101 (12)	−0.0034 (11)
C6	0.0422 (17)	0.073 (2)	0.059 (2)	0.0066 (14)	0.0209 (16)	0.0057 (16)
C7	0.0416 (17)	0.084 (2)	0.062 (2)	−0.0189 (15)	0.0156 (16)	−0.0033 (17)
C8	0.0464 (16)	0.0391 (15)	0.0406 (18)	0.0057 (11)	0.0136 (13)	−0.0044 (11)
C9	0.090 (3)	0.083 (2)	0.038 (2)	0.0103 (19)	0.0133 (19)	−0.0022 (16)

### Geometric parameters (Å, º)

**Table:**

Cu1—Cl1^i^	2.9242 (8)	C2—H2A	0.9600
Cu1—Cl1	2.2609 (7)	C2—H2B	0.9600
Cu1—Cl2	2.2521 (7)	C2—H2C	0.9600
Cu1—O1	2.637 (2)	C3—H3	0.9300
Cu1—N1	2.0182 (19)	C3—C4	1.390 (3)
Cu1—N3	2.036 (2)	C4—C5	1.462 (4)
O1—C5	1.205 (3)	C6—H6A	0.9700
O2—C5	1.330 (3)	C6—H6B	0.9700
O2—C6	1.449 (3)	C6—C7	1.492 (4)
N1—N2	1.330 (3)	C7—H7A	0.9600
N1—C4	1.335 (3)	C7—H7B	0.9600
N2—H2	0.80 (3)	C7—H7C	0.9600
N2—C1	1.347 (3)	C8—C9	1.441 (4)
N3—C8	1.123 (4)	C9—H9A	0.9600
C1—C2	1.495 (4)	C9—H9B	0.9600
C1—C3	1.360 (4)	C9—H9C	0.9600
			
Cl1—Cu1—Cl1^i^	86.62 (3)	H2A—C2—H2C	109.5
Cl1—Cu1—O1	103.64 (5)	H2B—C2—H2C	109.5
Cl2—Cu1—Cl1	92.05 (3)	C1—C3—H3	127.0
Cl2—Cu1—Cl1^i^	108.61 (3)	C1—C3—C4	106.1 (2)
Cl2—Cu1—O1	95.89 (5)	C4—C3—H3	127.0
O1—Cu1—Cl1^i^	153.19 (4)	N1—C4—C3	110.2 (2)
N1—Cu1—Cl1	89.44 (6)	N1—C4—C5	116.5 (2)
N1—Cu1—Cl1^i^	85.41 (6)	C3—C4—C5	133.2 (2)
N1—Cu1—Cl2	165.96 (7)	O1—C5—O2	124.1 (2)
N1—Cu1—O1	70.22 (7)	O1—C5—C4	123.3 (2)
N1—Cu1—N3	90.84 (8)	O2—C5—C4	112.6 (2)
N3—Cu1—Cl1	171.83 (7)	O2—C6—H6A	110.2
N3—Cu1—Cl1^i^	85.27 (7)	O2—C6—H6B	110.2
N3—Cu1—Cl2	89.66 (6)	O2—C6—C7	107.4 (2)
N3—Cu1—O1	84.12 (8)	H6A—C6—H6B	108.5
C5—O1—Cu1	104.76 (17)	C7—C6—H6A	110.2
C5—O2—C6	116.2 (2)	C7—C6—H6B	110.2
N2—N1—Cu1	128.73 (16)	C6—C7—H7A	109.5
N2—N1—C4	105.05 (19)	C6—C7—H7B	109.5
C4—N1—Cu1	124.95 (17)	C6—C7—H7C	109.5
N1—N2—H2	123.6	H7A—C7—H7B	109.5
N1—N2—C1	112.7 (2)	H7A—C7—H7C	109.5
C1—N2—H2	123.6	H7B—C7—H7C	109.5
C8—N3—Cu1	175.2 (2)	N3—C8—C9	179.8 (4)
N2—C1—C2	121.7 (2)	C8—C9—H9A	109.5
N2—C1—C3	105.9 (2)	C8—C9—H9B	109.5
C3—C1—C2	132.4 (2)	C8—C9—H9C	109.5
C1—C2—H2A	109.5	H9A—C9—H9B	109.5
C1—C2—H2B	109.5	H9A—C9—H9C	109.5
C1—C2—H2C	109.5	H9B—C9—H9C	109.5
H2A—C2—H2B	109.5		
			
Cu1—O1—C5—O2	178.2 (2)	N2—C1—C3—C4	−1.0 (3)
Cu1—O1—C5—C4	−0.4 (3)	C1—C3—C4—N1	1.1 (3)
Cu1—N1—N2—C1	167.66 (19)	C1—C3—C4—C5	−174.1 (3)
Cu1—N1—C4—C3	−168.89 (18)	C2—C1—C3—C4	177.7 (3)
Cu1—N1—C4—C5	7.2 (3)	C3—C4—C5—O1	171.4 (3)
N1—N2—C1—C2	−178.3 (3)	C3—C4—C5—O2	−7.4 (4)
N1—N2—C1—C3	0.5 (3)	C4—N1—N2—C1	0.2 (3)
N1—C4—C5—O1	−3.7 (4)	C5—O2—C6—C7	−179.7 (2)
N1—C4—C5—O2	177.6 (2)	C6—O2—C5—O1	−3.1 (4)
N2—N1—C4—C3	−0.8 (3)	C6—O2—C5—C4	175.7 (2)
N2—N1—C4—C5	175.3 (2)		

Symmetry code: (i) −*x*+1, −*y*+1, −*z*+2.

### Hydrogen-bond geometry (Å, º)

**Table:**

*D*—H···*A*	*D*—H	H···*A*	*D*···*A*	*D*—H···*A*
N2—H2···Cl1^i^	0.80 (2)	2.72 (2)	3.281 (2)	129 (2)
N2—H2···Cl2^i^	0.80 (2)	2.59 (2)	3.273 (2)	145 (1)
C7—H7*A*···Cl2^ii^	0.96	2.79	3.662 (4)	151

Symmetry codes: (i) −*x*+1, −*y*+1, −*z*+2; (ii) −*x*, *y*−1/2, −*z*+3/2.
